# Supplementary material for: Air-Assisted Electrospinning of Dihydromyricetin-Loaded Dextran/Zein/Xylose Nanofibers and Effects of the Maillard Reaction on Fiber Properties
Source: Molecules. 2024 Jul 1;29(13):3136. doi: 10.3390/molecules29133136 (PMC11243030; doi:10.3390/molecules29133136)
Supplement: Supplementary file 1 [file molecules-29-03136-s001.zip › molecules-3056822-supplementary.pdf]

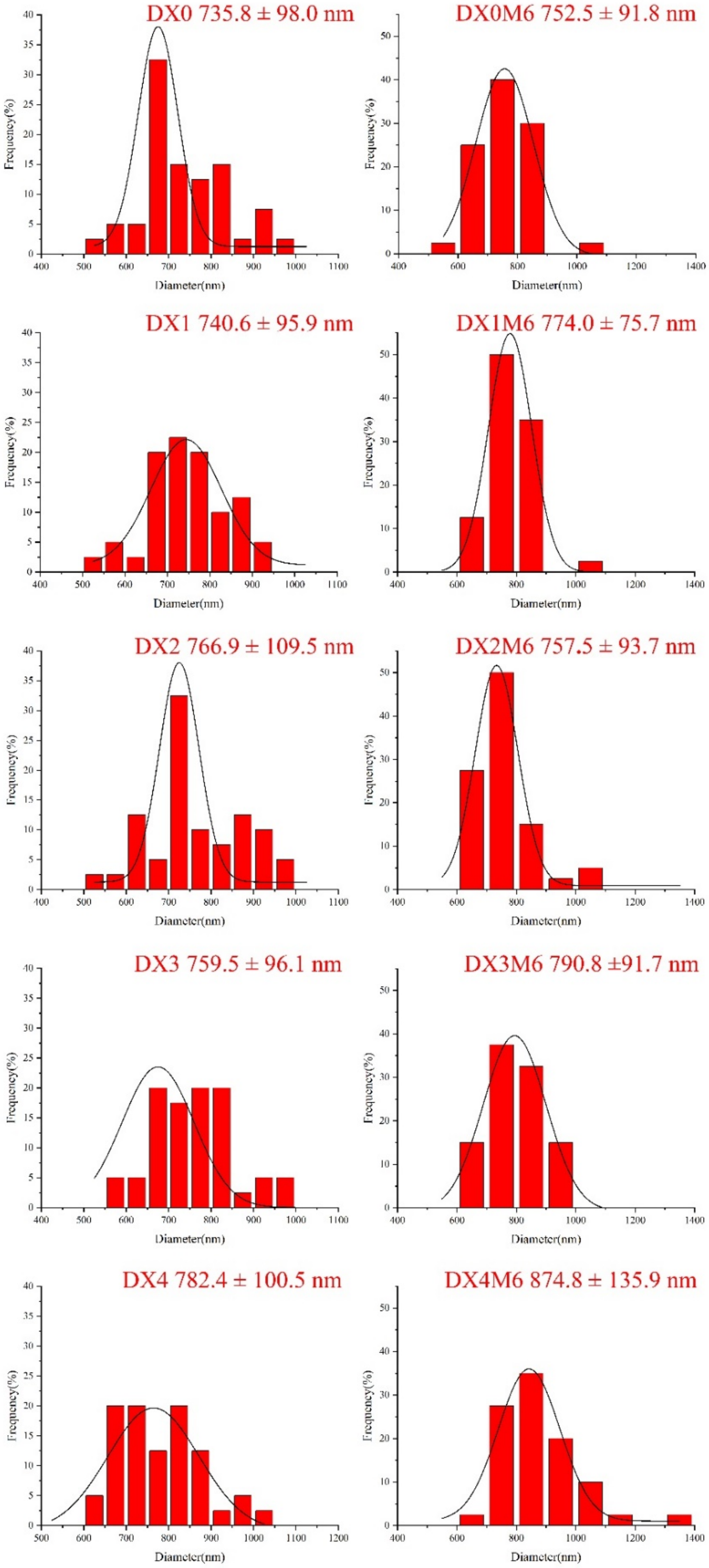

Figure S1. Diameter histograms of the dextran/zein nanofibers without (DX0) or with 1% (DX1), 2% (DX2), 3% (DX3), and 4% (DX4) dihydromyricetin, and those cross-linked for 6 h, which were denoted as DX0M6, DX1M6, DX2M6, DX3M6, and DX4M6, respectively.
